# The Prevalence, Etiologic Agents and Risk Factors for Urinary Tract Infection Among Spinal Cord Injury Patients

**DOI:** 10.5812/jjm.8905

**Published:** 2014-01-01

**Authors:** Turhan Togan, Ozlem Kurt Azap, Elif Durukan, Hande Arslan

**Affiliations:** 1 Department of Infectious Diseases and Clinical Microbiology, Faculty of Medicine, Baskent University, Ankara, Turkey; 2 Department of Public Health, Baskent University Faculty of Medicine, Ankara, Turkey

**Keywords:** Urinary Tract Infections, Spinal Cord Injury, Asymptomatic Bacteriuria, Symptomatic Bacteriuria

## Abstract

**Background:**

Urinary tract infections (UTIs) are important causes of morbidity and mortality in patients with spinal cord injury and 22% of patients with acute spinal cord injury develop UTI during the first 50 days.

**Objectives:**

The aim of this study was to determine the prevalence, etiologic agents and risk factors for asymptomatic bacteriuria and symptomatic urinary tract infections in patients with spinal cord injury.

**Patients and Methods:**

This was a prospective investigation of spinal cord injury patients with asymptomatic bacteriuria and symptomatic urinary tract infections in Baskent University Medical Faculty Ayas Rehabilitation Center and Ankara Physical Therapy and Rehabilitation Center between January 2008 and December 2010. The demographic status, clinical and laboratory findings of 93 patients with spinal cord injury were analyzed in order to determine the risk factors for asymptomatic or symptomatic bacteriuria

**Results:**

Sixty three (67.7%) of 93 patients had asymptomatic bacteriuria and 21 (22.6%) had symptomatic urinary tract infection. Assessment of the frequency of urinary bladder emptying methods revealed that 57 (61.3%) of 93 patients employed permanent catheters and 24 (25.8%) employed clean intermittent catheterization. One hundred and thirty-five (48.0%) of 281 strains isolated form asymptomatic bacteriuria attacks and 16 (66.6%) of 24 strains isolated from symptomatic urinary tract infection attacks, totaling 151 strains, had multidrug resistance (P > 0.05). One hundred (70.4%) of 142 *Escherichia coli* strains and 19 (34.5%) of 55 *Klebsiella* spp strains proliferated in patients with asymptomatic bacteriuria; 8 (80%) of 10 *E. coli *strains and 4 (80%) of 5 *Klebsiella *spp. strains were multidrug resistant.

**Conclusions:**

The most common infectious episode among spinal cord injury patients was found to be urinary tract ınfection. *E. coli *was the most common microorganism isolated from urine samples. Antibiotic use in the previous 2 weeks or 3 months, hospitalization during the last one-year and previous diagnosis of urinary tract ınfection were the risk factors identified for the development of infections with multi-drug resistant isolates. Urinary catheterization was found to be the only independent risk factor contributing to symptomatic urinary tract infection.

## 1. Background 

Urinary tract infections (UTIs) are important causes of morbidity and mortality in patients with spinal cord injury (SCI). It has been reported that 22% of patients with acute SCI develop UTI during the first 50 days and annual UTI incidence in patients with chronic SCI is nearly 20% (1-6). Mortality rate in individuals with spinal cord injury is as high as 6.3% in the first year after the injury, whereas it significantly decreases in the subsequent years (7).

Cause of mortality in individuals with SCI is mainly respiratory diseases with a rate of 21.7% while heart diseases rank second with a rate of 12.6% and infections rank third with a rate of 9.4% (8). While urinary system infections are major causes of mortality and morbidity in patients with SCI, mortality due to urinary sepsis has decreased to 10-15% with improved management (2).

## 2. Objectives

The aim of this study was to determine the prevalence and causative agents of asymptomatic bacteriuria (ASB) and symptomatic urinary system infection (SUSI), and risk factors for UTI in patients with SCI.

## 3. Patients and Methods

A total of 93 patients with SCI were followed at Baskent University Medical Faculty Ayas Rehabilitation Center and Ankara Physical Therapy and Rehabilitation Center between 15^th^ of February 2008 and 30^th^ of June 2009. Patients belonged to the age group of 18-65 years. Patients with SCI were prospectively where followed Subjects were visited every week. A form was filled for each patient; containing demographic features, clinical signs and symptoms, laboratory and imaging findings, isolated microorganisms and their sensitivities to antibiotics. Urinary samples and culture was taken from patients during each visit. Antibiotic sensitivities were determined by the Kirby-Bauer disk diffusion method in compliance with Clinical Laboratory Standards Institute (CLSI) rules (9).

The threshold level for significant bacteriuria was accepted as 10^5 ^cfu/mL (10). A patient was considered to have symptomatic urinary system infection if he/she had significant bacteriuria plus one of the following clinical findings; body temperature > 38°C, abdominal pain, reflex perspiration, urinary incontinence, increased spasticity, suprapubic pain, flank pain, frequent urination, dysuria, urgency, urinary incontinence, foul smell in urine, and cloudy urine (11-14). Patients with significant bacteriuria but no fever or clinical complaints were considered to have asymptomatic bacteriuria (5, 15). Patients with bacteriuria and fever but no urinary complaint, whose fever was due to another cause, were considered to have “non-UTI infection”. 

All patients were followed up with weekly complete blood counts and CRP tests. Eighty-four patients underwent abdominal ultrasonography to screen for trabeculations in urinary bladder (present/absent), parenchymal changes in kidney (present/absent), and vesicoureteral reflux (present/absent). Strains resistant to at least three of quinolones, beta-lactams, aminoglycosides, and co-trimoxazole group antibiotics were defined as “multi-drug resistant (MDR) strains”. 

### 3.1. Statistical Analysis

Study data were analyzed using Statistical Package for the Social Sciences (SPSS) 17.0 (SPPS Inc., Chicago, IL, USA) software. Categorical variables were compared by the Chi-Square and Fisher’s exact Chi-Square test; comparison of differences of continuous variables was carried out with t test in independent groups. Risk factors for bacteriuria were determined with logistic regression analysis. A patient value of less than 0.05 was considered significant.

## 4. Results

Ninety-three patients (78 males, 15 females) were included. Mean age was 35.65 ± 13.11. Spinal cord injury level was cervical in 30.5%, thoracic in 63%, lumbar in 6.5%. Seventy-three (78.5%) patients were paraplegic and 17 (18.3%) were quadriplegic. Three (3.2%) patients had minor spinal cord injury with associated paresthesia. The most common causes of spinal cord injury were traffic accidents in 44.1% and falls in 38.7%. Firearm wounds, trauma, vertebral mass, electric shock, and operation were other causes, in descending order. Mean duration of urinary catheterization was 2.5 months. A mean of 4.2 ± 1.2 visits, each one-week apart, took place. A total of 397 visits were paid to 93 patients. Bacteriuria was detected in 84 (90.3%) of 93 patients, while 9 (9.7%) had no proliferation. Sixty three (67.7%) of 93 patients had ASB and 21 (22.6%) had SUSI. Twenty-one patients with the diagnosis of SUSI developed 24 attacks and 63 patients with ASB developed 281 attacks, totaling 305 bacteriuria attacks. Twenty-seven of 93 patients developed 30 infectious attacks during the follow-up. Twenty-one patients had a total of 24 SUSI attacks and 6 patients had infections of other systems (4 respiratory system infections, 1 bacteremia and 1 dental abscess).

Assessment of the frequency of urinary bladder emptying methods revealed that 57 (61.3%) of 93 patients employed permanent catheters and 24 (25.8%) employed clean intermittent catheterization (CIC). Twelve (12.9%) patients were able to urinate spontaneously. ASB/SUSI development was assessed and it was found that 34 (59.6%) of 57 patients with permanent catheters developed ASB, 17 (29.8%) had SUSI, whereas 6 (10.6%) had no proliferation.

Twenty (83.3%) of 24 patients in whom clean intermittent catheterization was used, were diagnosed with ASB and 4 (16.7%) were diagnosed with SUSI. Three of 12 patients urinating spontaneously had no proliferation. There was no significant relationship between catheterization type and ASB or SUSI development.

Bladder emptying method in 63 followed patients with asymptomatic bacteriuria was with a permanent catheter in 34 (53.9%), CIC in 20 (31.7%), and spontaneous urination in 9 (14.4%). Seventeen (80.9%) of 21 patients followed up with symptomatic UTI had permanent catheters and 4 (19.1%) had CIC. No patients among those with SUSI urinated spontaneously. Six of 9 patients with no proliferation had permanent catheterization while 3 were able to urinate spontaneously.There was no significant difference between bladder emptying methods in terms of bacteriuria.

Agents isolated from ASB and SUSI attacks are mentioned in Table 1. 

**Table 1.** Distribution of Agents Isolated from Asymptomatic Bacteriuria and Symptomatic UTI Attacks

Microorganism	ASB, No. (%)	SUSI, No. (%)
***E. coli***	142 (50.5)	10 (41.7)
***Klebsiella spp.***	55 (19.6)	5 (20.8)
***Enterococcusspp.***	23 (8.2)	2 (8.3)
***Pseudomonasspp.***	15 (5.3)	2 (8.3)
**KNS ** ^**a**^	8 (2.9)	-
***Acinetobacterspp.***	7 (2.5)	3 (12.6)
***Proteus*** ** spp.**	6 (2.1)	-
***S.aureus***	4 (1.4)	-
***Candidaspp.***	4 (1.4)	2 (8.3)
***Enterobacterspp.***	2 (0.8)	-
**Other ** ^**b**^	15 (4.6)	-
**Total**	281 (100.0)	24 (100.0)

^a^ Coagulase negative staphylococci

^b^ *S.malthophilia, M.morganii, Corynebacterium* spp

There was no statistical difference between strains isolated from patients diagnosed with symptomatic UTI and ASB in terms of antibiotic resistance (Tables 2 and 3). Thirty (47.6%) from 63 patients were followed up for ASB, 11 (52.4%) of 21 patients were followed up for SUSI, and 4 (44.4%) of 9 patients with no proliferation had a history of antibiotic use in the last 3 months. Although antibiotic use was more prevalent in those with bacteriuria, there was no significant difference between the groups (P *> *0.05).

**Table 2.** Rates of Antibiotic Resistance of *E.coli* Strains Isolated From Symptomatic UTI and ASB Attacks.

Antibiotic	ASB, No. (%)^a^	SUSI, No. (%)^a^	P Value^b^
**Ampicillin**	124 (87.3)	8 (80.0)	>0.05
**Cefuroxime**	101 (71.1)	7 (70.0)	>0.05
**Co-Trimoxazole**	95 (66.9)	8 (80.0)	>0.05
**Ciprofloxacin**	87 (61.3)	6 (60.0)	>0.05
**Amoxicillin**	85 (59.9)	3 (30.0)	>0.05
**Ceftriaxone **	71 (50.0)	5 (50.0)	>0.05
**Gentamicin**	57 (40.1)	2 (20.0)	>0.05
**Cefepime**	29 (20.4)	0	>0.05
**Piperacillin-Tazobactam**	12 (8.5)	0	>0.05
**Amikacin**	7 (4.9)	0	>0.05
**Imipenem**	0	0	0
**Meropenem**	0	0	0

^a^ The percentage of resistant strains

^b^ Ki-Kare test

**Table 3.** Rates of Antibiotic Resistance of *Klebsiella *spp. Strains Isolated From Patients With Symptomatic UTI and ASB

Antibiotic	ASB No. (%)^a^	SUSI, No. (%)^a^	P Value ^ b^
**Co-Trimoxazole**	23 (41.8)	4 (80.0)	>0.05
**Cefuroxime**	22 (40.0)	3 (60.0)	>0.05
**Amoxicillin**	21 (38.2)	5 (100.0)	>0.05
**Ceftriaxone**	19 (34.5)	1 (20.0)	>0.05
**Ciprofloxacin**	16 (29.1)	3 (60.0)	>0.05
**Gentamicin**	12 (21.8)	2 (40.0)	>0.05
**Cefepime**	12 (21.8)	1 (20.0)	>0.05
**Piperacillin-Tazobactam**	4 (7.3)	2 (40.0)	>0.05
**Amikacin**	3 (5.5)	1 (20.0)	>0.05
**Imipenem**	0	0	
**Meropenem**	0	0	

^a^ The percentage of resistant strains

^b^ Ki-kare test

One hundred and thirty-five (48.0%) of 281 strains isolated form ASB attacks and 16 (66.6%) of 24 strains isolated from SUSI attacks, totaling 151 strains, had multidrug resistance (*P *> 0.05). One hundred (70.4%) of 142 *E*. *coli *strains and 19 (34.5%) of 55 *Klebsiella. spp* strains proliferated in patients with asymptomatic bacteriuria; 8 (80%) of 10 *E.coli strains and 4 (80%) of 5 Klebsiella *spp*. *strains were multidrug resistant. The ratio of strains, which were multidrug resistant, was significantly higher in patients diagnosed with SUSI compared to those diagnosed with ASB (*P *< 0.05).

Nineteen (55.8%) of 34 patients from whom multidrug resistant bacteria were isolated had a history of antibiotic use in the last 3 months, 14 (47.1%) had a history of hospitalization within the last year, and 13 (38.2%) had a history of UTI. Leukocytosis was detected in 30 (14.1%) of 281 ASB attacks and 8 (38.1%) of 24 SUSI attacks. The likelihood of SUSI in patients with leukocytosis was 3.95 times greater (OR = 3.95; 95% CI = 1.49-10.51). CRP elevation was detected in 88 (57.9%) of 281 ASB attacks, whereas 11 (84.6%) of 24 SUSI attacks had CRP elevation (*P *> 0.05). Pyuria was present in 92 (44%) of 281 patients with ASB attack, 11 (55%) of 24 patients with SUSI. (P < 0.05). Nitrite positivity in urine was present in 131 (62.4%) of 281 ASB attacks and 14 (70%) of 24 SUSI attacks (P > 0.05).

Eighty-four patients underwent urinary ultrasonography and 77 (91.7%) had normal results whereas 7 (8.3%) had urinary system pathologies (nephrolithiasis, vesicoureteral reflux, benign prostate hypertrophy, nephrectomy). Three (3.6%) patients had urinary stones. Forty-one (48.8%) from a total of 84 patients with bacteriuria and 4 (44.4%) of 9 patients without bacteriuria had a history of hospitalization within the previous year. Twenty-seven (42.8%) of 63 followed patients with ASB and 10 (47.6%) of 21 followed patients with SUSI had a history of hospitalization within the previous year. Those with bacteriuria had a greater rate of hospitalization; albeit statistically insignificant (P >0.05).

Thirty-seven (44%) of 84 patients had a history of SUSI within the previous year. 4 (44%) of 9 patients with no bacteriuria had a history of SUSI within the previous year. Forty-six (54.7%) of 84 patients had a history of antibiotic use in the last 3 months while 4 (44.4%) of those 9 without bacteriuria had a similar history. The most commonly used antibiotic class was quinolones (Table 4). 

**Table 4.** Antibiotics Used in the Last 3 Months

Antibiotic	Patient, No. (%)
**Quinolone**	15 (32.6)
**Co-Trimoxazole**	11 (23.9)
**Cephalosporin**	7 (15.2)
**Penicillin **	6 (13.0)
**Carbapenem**	3 (6.5)
**Other^a^**	4 (8.7)
**Total**	46 (100.0)

^a^ Fluconazole, Nitrofurantoin

The mean interval from admission to SUSI attack was 22.20 ± 15, 73 days. There was no significant difference between spinal cord injury levels in terms of having SUSI (P > 0.05). There was no significant difference between genders in terms of having SUSI (P > 0.05). SUSI was not related to gender, age, duration or level of injury, duration of catheterization, having UTI or hospitalization within the previous year, antibiotic use within the last 3 months, presence of decubitus ulcers, presence of stones, increased CRP levels, presence of pyuria, nitrite positivity, presence of trabeculations, or renal parenchymal changes observed at USG.

## 5. Discussion

A total of 93 patients, diagnosed with SCI and treated at physical therapy and rehabilitation units, were included in this study. Percentage of males was 83.9, with a male-to female ratio similar to world data. In United States of America, approximately 18-33% of patients with SCI were female and 67-82% were male (16, 17). Mean age for SCI has been reported as 37 ± 11.5 years worldwide (18, 19) and 35.9 years in the United States (8). Turkish studies have reported a mean age of 35.5 years (20). Mean age of our patients was 35.65 ± 13.11 years, which was similar to the world average.

Eighty-four (90.3%) of 93 patients in our study had bacteriuria whereas 9 (9.7%) had no proliferation. Ruz et al. reported that the incidence of bacteriuria was 2.72 attacks/100 patient days patient hospitalization days and for UTI this was0.68 attack/100 patient days patient hospitalization days in patients for whom a urinary drainage method was employed (21). Twenty-five percent of 84 patients with bacteriuria had a SUSI attack and 75% had an ASB attack. Fifty-seven (61.3%) of 93 patients used a permanent catheter, 24 (25.8%) used a CIC. Twelve (12.9%) patients were able to urinate spontaneously. Six (66.6%) of 9 (9.7%) patients with no proliferation were followed with a permanent catheter, 3 (33.4%) of them were able to urinate spontaneously. These patients did not experience significant bacteriuria during their hospital stay despite prolonged catheterization.

Oz et al. in a study including 63 patients with SCI found that the rate of bacteriuria was 53.3% in patients using CIC and 82.9% in those with permanent catheters (22). Ruz et al. in a study examining 128 patients with SCI, reported that the rate of bacteriuria was 5 attacks/100 patient days patient hospitalization days, 2.95 attacks/100 patient days patient hospitalization days, 2.41 attacks/100 patient days patient hospitalization days, and 0.96 attack/100 patient days patient hospitalization days in permanent catheterization, clean intermittent catheterization, and condom catheter in males and supra pubic catheterization in females, respectively (21). The same study reported a bacteriuria rate of 0.33 attack/100 patient days patient hospitalization days in incomplete injuries with normal function of urination (21). The highest bacteriuria rate was due to permanent catheterization while the lowest rate was observed in those who were able to urinate. The finding of a higher bacteriuria rate in permanent catheterization compared to CIC is in agreement with the literature.

In a study from Portugal, 24.6% of catheterized patients were detected to have SUSI. It was also observed that UTI was more frequent in patients with a permanent catheter compared to those employing CIC (21). Oz et al. reported a SUSI rate of 61.5% in those with a urinary catheter (22). The highest UTI rate was observed in those with a permanent catheter. Our finding of a higher SUSI attack rate in permanent catheter users compared to CIC users is consistent with the literature. Microorganisms commonly isolated from urine cultures of patients with SCI are *E.coli, Pseudomonas* spp., *Klebsiella *spp., *Proteus* spp., *Serratia* spp., *Providencia* spp., *Enterococci*, and *Staphylococci* (23-25). Inspection of 305 agents isolated from a total of 84 patients followed up for bacteriuria revealed that the most common agent was *E.coli* with a percentage of 49.9%, followed by (in descending order) *Klebsiella* spp. (19.7%), *Enterococcus* spp. (8.2%), and *Pseudomonas *spp.(5.6%).

In our study, agents responsible for SUSI in SCI patients were *E. coli *in 41.7%, *Klebsiella spp.* in 20.8%, and *Acinetobacter* spp. in 12.6%. *E. coli* (50.5%) was the most commonly isolated pathogen in patients with ASB. As one can observe, *E. coli* takes the top place in agent distribution in patients followed up with SUSI and ASB. There was no significant difference between the distribution of agents isolated from patients with asymptomatic bacteriuria and patients with symptomatic urinary system infection.

Yadav et al. reported that* K. pneumonia* and *P. aeruginosa* proliferated in urine cultures in 75% of patients when CIC was administered during early stages of SCI. In addition they reported that the most commonly isolated organism was *E. coli* in cases with a prolonged follow-up (26). Polymicrobial infection comes into question with prolonged catheterization, which may give rise to proliferation of rare agents such as *Morganella spp.* and *Providencia* spp .(27). Despite the excellent care of patients catheterized for a long time, bacteriuria is inevitable. Both new bacteriuria episodes and persistent bacteriuria caused by some bacteria species may last for weeks or even months (12, 28).

In spinal cord injury, UTI agents are generally polymicrobial (1). In a study by Dedeic-Ljubovi et al. UTI attacks were generally polymicrobial while 44% had single bacteria species isolated (29). Since the significant proliferation threshold in our study was assumed as 10^5 ^cfu/mL, we did not observe a polymicrobial bacteriuria attack (10). Some studies consider 10^5 ^cfu/mL as the threshold value for bacteriuria while many studies consider proliferations at 10^2 ^cfu/mL significant since such proliferations increase in subsequent days (30). Since proliferations at the level of 10^2^cfu/mL have been considered abnormal, rates of polymicrobial bacteriuria in our study were inconsistent with the literature. 

We studied antibiotic sensitivities of isolated agents to guide empiric antimicrobial treatment of SUSI (31). Among isolated strains, 61.2% of *E. coli*, and 31.7% of *Klebsiella* *spp. *strains were resistant to ciprofloxacin, 67.8% of *E. coli* and 45.0% of *Klebsiella* *spp. *were resistant toco-trimoxazole, 50.0% of *E. coli* and 33.3% of *Klebsiella spp. *were resistant to ceftriaxone, and 38.8% of *E. coli* and 23.3% of *Klebsiella spp.* were resistant to gentamicin. All isolated strains were sensitive to carbapenems. According to our results we defined that the efficiency of ciprofloxacin has been decreased but carbapenems has been effective in treatment of urinary system infections.

As studies of patients with spinal cord injury have been sparse, data on antimicrobial sensitivity are also quite limited. In a thesis study from Trakya University Medical Faculty in 1995, all 10 *E. coli *strains isolated from patients with urinary catheters were sensitive to amikacin, carbapenems, and quinolones, where as 70% were resistant to co-trimoxazole (32). One study from Portugal reported that quinolone resistance has been increasing in bacteria isolated from catheter-related UTI attacks and majority of strains were more sensitive to amoxicillin than quinolones (33). Our results, along with other studies, show that sensitivity to quinolone and co-trimoxazole has been decreasing.

It has been reported that 2-4% of patients with spinal cord injury who are urinary catheterized have bacteremia (34, 35). Bacteremia attacks in our study were independent of SUSI. Falknier (36) reported that bacteremia prevalence was high in patients with prolonged catheterization. Our data on bacteremia were not consistent with the previous literature. This is possibly secondary to the small sample size and lack of blood cultures taken at febrile periods. Difficult-to-cure multidrug-resistant bacteria also complicate UTI therapy. In a study by Dedeic-Ljubovi et al. 55.3% of 3963 strains, isolated from patients with spinal cord injury who were catheterized, were multidrug-resistant. 87.8% of *A. baumannii* strains, 86.7% of* P. rettgeri* strains, 85.4% of* P. aeruginosa* strains, 84.3% of* P. stuarti* strains, and 81% of* M.* *morganii* strains were multidrug-resistant (29).

There were no significant difference between two groups (135 (48.0%) of 281 strains isolated from ASB attacks and 16 (66.6%) of 24 strains isolated from SUSI attacks,) (P > 0.05). We defined multidrug resistance as resistance to at least three of the quinolones, beta-lactams, and aminoglycosides groups and obtained a high rate of multidrug-resistance, consistent with the literature. Among patients from whom multidrug-resistant bacteria were isolated, 44.1% had a history of using antibiotics in the previous 2 weeks, 55.8% had used antibiotics within the last 3 months, 47.1% were hospitalized in the previous year, and 38.2% had had UTI within the last year. These rates were not statistically significant but recent antibiotic use, history of hospitalization or UTI within the previous 1 year should suggest that an infection has developed by multidrug-resistant bacteria. This is because nearly half of the patients from whom multidrug-resistant bacteria were isolated had these factors, albeit statistically non-significant.

In 91 of 93 patients in our study, body temperature was 37.5^o^C or below. Two of the patients followed-up for symptomatic UTI had a body temperature of 38-40^o^C, which dropped with treatment. Therefore, there was no significant relationship between SUSI attack and body temperature. Mean SCI level was T6 or higher. This condition may suggest a tendency towards hypothermia due to the possible alteration of thermoregulatory system response. Studies have shown that 32-40% of patients with SCI had fever during their UTI attack (37, 38). In the study by Oz et al. CRP was high in 44% of SCI patients with UTI (22). In our study, on the other hand, CRP elevation was present in 57.9% of ASB attacks and 84.6% of SUSI attacks. Both studies have not found any significant relationship between increased CRP level and UTI development.

While Oz et al. detected leukocytosis in 19% of UTI cases (22), we found leukocytosis in 14.1% of ASB attacks and 38.1% of SUSI attacks. Likelihood of SUSI in patients with leukocytosis was 3.95 times greater. Pyuria in our study was present in 44% of patients with ASB and 55% of those with SUSI. Presence of SUSI was not a significant predictor of SUSI. One study demonstrated that pyuria was associated with bacteriuria with a sensitivity of 74% and specificity of 95.9% (39). However, presence of pyuria is not a sufficient criterion for the diagnosis of bacteriuria. Furthermore, it does not distinguish symptomatic versus asymptomatic urinary system infection since other inflammatory conditions of genitourinary system may cause pyuria (5). Deresinski and Perkash, in a study of 70 patients with SCI, reported that bacteriuria was accompanied by pyuria in 97.4% of patients, whereas 40.6% of patients were asymptomatic despite significant pyuria (39). Oz, unlike these results, detected pyuira as the sole indicator for the development of UTI in patients with SCI (22).

Abdominal ultrasonographic examinations of patients with SCI, have indicated that urinary bladder pathologies were more common in males and in those with a higher level of injury, longer disease duration, and complete injury. Bladder stones and bladder trabeculations were reported in 2% and 1.8% of patients with SCI, respectively (40). Oz et al. detected renal parenchymal changes on urinary USG examination in 9.5% of patients. Investigation of the presence of stones and renal parenchymal changes revealed that 10 patients out of 46 had bladder trabeculations and 4 had renal parenchymal changes (22). Eighty-four patients in our study underwent urinary ultrasonographic examination and 3 (3.6%) had nephrolithiasis. This ratio was consistent with the literature. 

Ruz et al. defined risk factors for symptomatic UTI development in patients with spinal cord injury. They concluded that the following were risk factors for symptomatic UTI: level of cervical lesion, invasive interventions, and a urinary catheterization lasting longer than 30 days (21). We periodically monitored 93 patients for 18 months and a total of 397 visits were performed. The single independent risk factor for development of symptomatic UTI was catheterization. We found that 47.6% of patients with UTI had a history of antibiotic use within the last 2 weeks and 52.4% within the last 3 months, 47.6% had been hospitalized within the last year and 38.1% had had UTI within the last year. Many studies have defined these findings as risk factors. We think that we could not identify them as independent risk factors because of our limited sample size.
